# Gangliocytic paraganglioma: a multi-institutional retrospective study in Japan

**DOI:** 10.1186/s12885-015-1308-8

**Published:** 2015-04-12

**Authors:** Yoichiro Okubo, Tetsuo Nemoto, Megumi Wakayama, Naobumi Tochigi, Minoru Shinozaki, Takao Ishiwatari, Kyoko Aki, Masaru Tsuchiya, Hajime Aoyama, Kanade Katsura, Takeshi Fujii, Takashi Nishigami, Tomoyuki Yokose, Yasuo Ohkura, Kazutoshi Shibuya

**Affiliations:** 1 Department of Surgical Pathology, Toho University School of Medicine, 6-11-1 Omori-Nishi, Ota-ku, Tokyo, 143-8541 Japan; 2 Division of General and Gastroenterological Surgery, Department of Surgery (Omori), Toho University School of Medicine, 6-11-1 Omori-Nishi, Ota-ku, Tokyo, 143-8541 Japan; 3 Department of Pathology and Oncology, University of the Ryukyus, 59, Nishihara-cho, Nakagami-gun, Okinawa 903-0214 Japan; 4 Department of Pathology, Japanese Red Cross Kyoto Daini Hospital, 355-5, Jokyo-ku, Kyoto, 602-8026 Japan; 5 Department of Pathology, Toranomon Hospital, 2-2-2 Toranomon, Minato-ku, Tokyo, 105-8470 Japan; 6 Department of Pathology, Steel Memorial Hirohata Hospital, 3-1, Himeji, Hyogo 671-1122 Japan; 7 Department of Pathology, Kanagawa Cancer Center, 1-1-2, Nakao, Asahi-ku, Yokohama, Kanagawa 245-0815 Japan; 8 Department of Pathology, Kyorin University School of Medicine, 6-20-2, Shinkawa, Mitaka, 181-8611 Japan; 9 Department of Dermatology, Peking University First Hospital, Beijing, China

**Keywords:** Gangliocytic paraganglioma, Neuroendocrine tumor, Progesterone receptor, Pancreatic polypeptide

## Abstract

**Background:**

Gangliocytic paraganglioma (GP) is an extremely rare benign tumor that commonly arises from the second part of the duodenum. Since GP exhibit neither prominent mitotic activity nor Ki-67 immunoreactivity, this tumor is often misdiagnosed as neuroendocrine tumor (NET) G1 (carcinoid tumor). However, patients with GP may have a better prognosis than patients with NET G1. This fact emphasizes the importance of differentiating GP from NET G1, but few studies have reported the epidemiology and histopathology of GP because of its rarity. To differentiate GP from NET G1 with ease, we conducted a multi-institutional retrospective study analyzing the morphometric and immunohistochemical features of this tumor.

**Methods:**

Since only a limited number of patients with GP could be identified in our institute, we conducted a multi-institutional retrospective study of GP in Japan, which was approved by the Ethics Committee of our medical institute. The obtained tissue sections underwent detailed morphometric and immunohistochemical analyses. Additionally, to differentiate GP from NET G1 with ease, immunohistochemical findings were compared.

**Results:**

In our examination of 12 cases of duodenal GP, we found that epithelioid cells of GP exhibited positive reactivity for progesterone receptor and pancreatic polypeptide, whereas tumor cells of NET G1 were completely negative reactivity for both. Additionally, although GP is considered to be an extremely rare NET, we found that four (40.0%) of the ten patients at our institute with duodenal NET G1 actually had GP.

**Conclusions:**

Although GP is regarded as a rare NET, our results suggest that it accounts for a substantial percentage of duodenal NETs. Additionally, confirmation of immunoreactivity for progesterone receptor and pancreatic polypeptide can assist in differentiating GP from NET G1.

## Background

Gangliocytic paraganglioma (GP) is an extremely rare neuroendocrine tumor (NET) that commonly arises from the second part of the duodenum [1,2]. Histopathological diagnosis of GP requires confirmation of the following three characteristic tumor components: epithelioid, spindle-shaped, and ganglion-like cells. We previously elucidated the characteristics of GP in accordance with the Preferred Reporting Items for Systematic Reviews and Meta-Analyses [3]. Our investigation suggested that patients with this tumor have a good prognosis, and that neither irradiation nor chemotherapy is required for patients without residual tumor after surgical intervention [4]. However, standard clinical management of GP has not been established because of its rarity.

Meanwhile, in 2010 the World Health Organization (WHO) updated their classification of NET arising from the digestive system [5,6]. This WHO classification proposed a grading system for NET based on the proliferative activity of tumor cells, which is defined by the number of mitoses confirmed per 10 high-power microscopic fields, or, by the percentage of tumor cells showing positive reactivity for the Ki-67 antigen (the Ki-67 labeling index). Specifically, NET has been classified as grade 1 (G1: low grade, so-called carcinoid tumor), grade 2 (G2: intermediate grade), and grade 3 (G3: high grade).

Unfortunately, epithelioid cell component of GP has often been misdiagnosed as NET G1, because it exhibits neither prominent mitotic activity nor Ki-67 immunoreactivity [4]. However, the 5-year survival rate for patients with NET G1 has been reported to be approximately 80–90% [7]. There is no observational clinical study of patients with GP, regrettably; however, there is only one reported death from GP [4,8]. This indicates that the prognosis of GP is better than for NET G1. This difference in prognosis emphasizes the importance of differentiating GP from NET G1. Thus, to differentiate GP from NET G1 with ease, we carried out careful histopathological analyses in a multi-institutional retrospective study.

## Methods

### Collection of gangliocytic paraganglioma cases

#### Sample collection from our institute

As GP is a rare NET that commonly arises from the duodenum, we searched for cases of duodenal NET that were recorded between January 2000 and August 2013 using pathologic diagnosis support software (‘Dr. Helper’ System, JR West Japan Railway Company, Osaka, Japan). Specifically, we conducted searches for ‘carcinoid’ , ‘neuroendocrine’ , ‘karuchinoid’ (the Japanese word for carcinoid), and ‘shinkeinaibunpi’ (the Japanese word for neuroendocrine). During our search, the term ‘juunishicho’ (the Japanese word for duodenum) was used as an additional option to identify the tumor site. Subsequently, we examined tissue sections from the identified patients and defined their tumors as GP if three characteristic tumor components (epithelioid, spindle-shaped, and ganglion-like cells) in tissue sections were confirmed. Additionally, we extracted data from these patients for examination, including clinicopathological findings such as age, sex, operative procedure, lymph node metastasis status, and outcome.

#### Sample collection from other institutions

In April 2012, we searched for Japanese cases of GP using the Igaku Chuo Zasshi database (http://www.jamas.or.jp/). The search was specifically conducted using the terms ‘gangliocytic paraganglioma’ and ‘shoreihoukoku’ (the Japanese word for case report) was used as an additional option. We reviewed the selected publications to identify the authors’ contact information (all publications were reported in Japanese and not indexed by PubMed). We contacted the authors and explained the outline of this study. With their permission, we obtained clinicopathological data (age, sex, operative procedure, lymph node metastasis status, and outcome) and tissue sections of the tumors were mounted on silane-coated glass slides.

Before the use of these materials, this study was approved by the Ethics Review Committee of the Toho University School of Medicine, Tokyo, Japan (Approval Number: 23021).

### Histopathological examination of gangliocytic paraganglioma

Tissue sections were prepared and subjected to hematoxylin and eosin (H&E) staining for observation under a light microscope. Antibodies against the following were then used via immunohistochemically evaluating the three tumor cell types: Bcl-2 (1:50 dilution; Dako Japan, Tokyo, Japan, Clone name: 124), CD56 (1:100 dilution; Novocastra Newcastle upon Tyne, UK, Clone name: 1B6), chromogranin A (1:800 dilution; Dako Japan, Clone name: DAK-A3), estrogen receptor (Ready to Use; Roche Diagnostics Co., Tokyo, Japan, Clone name: SP1), Ki-67 (1:200 dilution; Dako Japan, Clone name: MIB-1), pan-cytokeratin (1:400 dilution; Dako Japan, Clone name: AE1/AE3), pancreatic polypeptide (1:100 dilution; Abcam, Cambridge, UK, incubated with Histofine Simple Stain MAX-PO (G) (Nichirei Bioscience, Tokyo, Japan), polyclonal), progesterone receptor (Ready to Use; Roche Diagnostics Co., Clone name: 1E2), somatostatin (Ready to use; Dako Japan, polyclonal), synaptophysin (1:40 dilution; Dako Japan, Clone name: M0776), and S-100 protein (1:2400 dilution; Dako Japan, polyclonal).

### Morphometric analysis of gangliocytic paraganglioma

It has been reported that even among GP, the distribution and population of the three cell types varies in each case [9]. Therefore, we employed morphometric analysis of GP to objectively elucidate the characteristics of each GP case examined. Namely, tumor cells per unit area were counted for each cell type in each patient. To obtain these cell counts, histopathological images of the tumor site were captured using a video microscope camera (DP70, Olympus, Tokyo, Japan). Epithelioid, spindle-shaped, and ganglion-like cells were manually counted in 50 random high-power fields (HPFs) of histopathological images. Additionally, previous investigators have suggested that epithelioid cells originate from the endoderm, but spindle-shaped and ganglion-like cells originate from the neuroectoderm [10]. To verify this hypothesis, we investigated correlations between the three characteristic components.

### Comparison of gangliocytic paraganglioma with duodenal neuroendocrine tumor grade 1

According to the WHO classification of NET [11], both GP and NET G1 belong to the “neuroendocrine neoplasms of the amupullary region”, but GP is distinguished from NET G1 and the ICD-O codes from them are different (GP: 8683/0, NET G1: 8240/3). Therefore, we regarded GP as a different entity from NET G1 in the present study. To differentiate GP from NET G1, the morphological and immunohistochemical findings of GP were compared with those of NET G1. Because the duodenum is the most common primary site of GP and most cases pursue benign course, six patients with duodenal NET G1 were used for our comparison (patients with NET G1 have the best prognosis, compared with patients with typical NETs [7]). To confirm the mitotic count and Ki-67 labeling index of duodenal NET G1, duodenal NETs that had been surgically removed at our institution were examined. Following the WHO classification system, each duodenal NET with fewer than two mitoses per ten HPFs and a Ki-67 labeling index less than 2% was defined as NET G1, but NETs containing epithelioid, spindle-shaped, and ganglion-like cells were defined as GP in the present study. To evaluate immunohistochemical differences between GP and duodenal NET G1, the same antibodies were used.

### Statistical analysis

The percentages of the three tumor cells were calculated for each patient and correlations were analyzed using Pearson’s product-moment correlation coefficient. Differences were considered significant at *P* < 0.05. The differences of positive rates for each immunohistochemistry marker between the epithelioid cells of GP (the major component of GP) and NET G1 were analyzed using chi-square test. All statistical analyses were performed using IBM SPSS Statistics version 20 (IBM Corp., Armonk, NY, USA).

## Results

### Identifying patients with gangliocytic paraganglioma

Our search identified 52 patients with duodenal NET who were treated at Toho University Omori Medical Center, Tokyo, Japan, between January 2000 and August 2013. Of these, we focused on 17 patients with duodenal NET, as NET was not histopathologically confirmed in the remaining 35 patients. Of the 17 patients with duodenal NET who were examined, 11 underwent endoscopic or open surgical resection that resulted in the final diagnosis. Ten of the 11 patients exhibited fewer than two mitoses per 10 HPFs and a Ki-67 labeling index of less than 2%. Four of the 10 patients were diagnosed with GP on the basis of the presence of the three characteristic components. Additionally, our multi-institutional retrospective study identified a further eight patients with GP. Therefore, in this study, we examined a total of 12 patients with duodenal GP.

### Clinical findings of duodenal gangliocytic paraganglioma

In this study, GP arose from the duodenum in all patients. Among these 12 patients with GP, patient ages ranged from 49 to 78 years (mean ± standard deviation (SD): 66.4 ± 7.9 years) at diagnosis. Nine patients were men and three were women. Seven patients underwent an endoscopic procedure to remove the tumor, and the remainder underwent open surgical resection. The follow-up period ranged from 6 to 91 months and neither recurrence nor death in patients with GP occurred. These findings are summarized in Table 1.

**Table 1:** **Clinicopathological findings of the collected duodenal gangliocytic paraganglioma**

Cases	Age (years)	Sex	Operation	Duodenal site	size (mm)	Depth	Lymph node metastasis	Outcome (months)
1	49	Male	Endoscopic resection	NOS	10	sm	Nagative	NED 84
2	61	Male	PPPD	Papilla of Vater	30	mp	Positive (station 8a)	NED 48
3	63	Male	Endoscopic resection	Papilla of Vater	22	oddi	Nagative	NED 12
4	63	Male	Endoscopic resection	Horizontal portion	15	sm	Nagative	NED 20
5	64	Male	PPPD	Papilla of Vater	42	sm	Nagative	NED 6
6	64	Female	Endoscopic resection	Papilla of Vater	7	sm	Nagative	NED 91
7	66	Male	Endoscopic resection	Papilla of Vater	12	oddi	Nagative	NED 90
8	67	Male	PPPD	Papilla of Vater	21	sm	Nagative	NED 12
9	72	Male	Local excision	NOS	40	mp	Nagative	NED 24
10	74	Female	PD	Second part	23	oddi	Positive	
(station 13)	NED 6							
11	76	Female	Endoscopic resection	Papilla of Vater	9	oddi	Nagative	NED 25
12	78	Male	Endoscopic resection	Papilla of Vater	12	oddi	Nagative	NED 84

*PD*: pancreatoduodenectomy, *PPPD*: pylorus-preserving pancreaticoduodenectomy, *NOS*: not otherwise specified, size: maximum diameter of the tumor, depth: depth of the tumor, sm: submucosal layer, oddi: sphincter oddi, mp: muscularis propria, *NED*: no evidence of disease.

Legend: In this study, a total of 12 patients with duodenal gangliocytic paraganglioma were collected and examined. Clinicopathological findings of the 12 patients are summarized.

### Histopathological findings of duodenal gangliocytic paraganglioma

Maximum tumor diameter ranged from 7 to 42 mm (mean ± SD: 20.3 ± 11.8 mm). Ten patients had GP localized within the submucosal layer or sphincter of Oddi, while two patients had GP invading the muscularis propria. Moreover, two patients had lymph node metastasis (Table 1).

**Figure 1:**
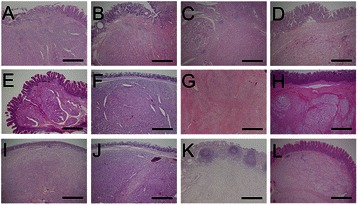
Histopathological findings of gangliocytic paraganglioma in the collected 12 cases of duodenal gangliocytic paraganglioma (low-power field). Legend: Photomicrographs showing low-power field of 12 cases of duodenal gangliocytic paraganglioma (GP). Even among patients with GP, histopathological findings varied widely microscopically. Most frequently, GP exhibited nested and compactly arranged epithelioid cells with scant stroma (Panel **A**, **B**, **D**, **E**, **F**, **H**, **I**, **J**, and **L**; **H & E** staining; magnification: × 40, scale bar represents 100 μm). In contrast, some GPs exhibited relatively sporadic nests of epithelioid cells and a predominance of stromal cells (Panel **C**, **G**, and **K**; **H & E** staining; magnification: × 40, scale bar represents 100 μm). Panel **A** to **L** correspond to Case 1 to 12 of GP.

**Figure 2:**
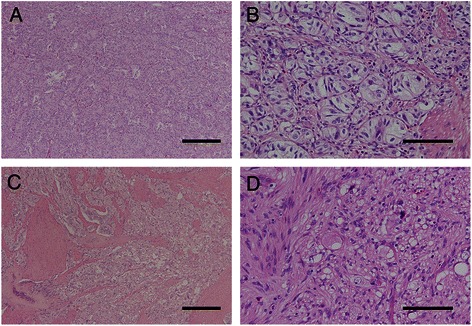
Representative histopathological findings of gangliocytic paraganglioma. Legend: **(A)** Photomicrograph showing a high-power field of the gangliocytic paraganglioma site featuring a dense proliferation of epithelioid cells. Nested and compactly arranged epithelioid cells comprise the majority of the tumor components (H&E staining; magnification: × 100, scale bar represents 300 μm). **(B)** Photomicrograph showing a low-power field of the gangliocytic paraganglioma site. Epithelioid cells had round to oval-shaped nuclei, inconspicuous nucleoli, and eosinophilic cytoplasm. Spindle-shaped cells surrounded the nests of epithelioid cells and were aligned in a single layer (H&E staining; magnification: × 400, scale bar represents 100 μm). **(C)** Photomicrograph showing a high-power field of the gangliocytic paraganglioma site revealing sporadic proliferation of epithelioid cells. A chaotic arrangement of epithelioid cells and a predominance of stromal cells, (e.g., smooth muscle cells, vessels, lymphoid follicles, fibrous tissue, and spindle-shaped cells) were confirmed (H & E staining; magnification: × 100, scale bar represents 300 μm). **(D)** Epithelioid cells showed a random arrangement, and spindle cells in the stroma were arranged in an irregular pattern (H&E staining; magnification: × 400, scale bar represents 100 μm).

Although all patients were diagnosed with GP, histopathological findings varied widely between patients (Figure 1). Most frequently, GP exhibited nested and compactly arranged epithelioid cells, with round to oval-shaped nuclei, inconspicuous nucleoli, and clear and eosinophilic cytoplasm. In these patients, scanty stroma was confirmed and spindle-shaped cells that surrounded the nests of epithelioid cells were aligned in a single layer (Figure 2A and B).

In contrast, some patients with GP exhibited relatively sporadic nests of epithelioid cells and a predominance of stromal cells (e.g., smooth muscle cells, vessels, lymphoid follicles, fibrous tissue, and spindle-shaped cells). In these patients, the percentage of spindle cells in the stroma varied widely. However, in all patients with GP, ganglion-like cells were rarely observed (Figure 2C and D).

### Morphometric analysis of gangliocytic paraganglioma

Even among patients with GP, the distribution of the three characteristic tumor cells varied from case to case. The counts of epithelioid, spindle-shaped, and ganglion-like cells in 50 random HPFs of histopathological images from each patient ranged from 3004 to 18,082 (mean ± SD: 10,396.3 ± 4804.9), 1372 to 6426 (mean ± SD: 2896.5 ± 1472.2), and 3 to 44 (mean ± SD: 14.8 ± 13.1), respectively. The percentages of epithelioid, spindle-shaped, and ganglion-like cells in 50 random HPFs of histopathological images from each patient ranged from 37.11 to 91.65% (mean ± SD: 75.50 ± 15.04%), 8.33 to 62.47% (mean ± SD: 24.37 ± 14.94%), and 0.01 to 0.43% (mean ± SD: 0.13 ± 0.12%), respectively (Table 2).

**Table 2:** **The counts and percentages of each of the three characteristic tumor cells**

	Epithelioid cells	Spindle-shaped cells	Ganglion-like cells	total
Case 1	18082 (89.93%)	2022 (10.06%)	3 (0.01%)	20107
Case 2	9271 (69.97%)	3976 (30.00%)	3 (0.02%)	13250
Case 3	3817 (37.11%)	6426 (62.47%)	44 (0.43%)	10287
Case 4	11318 (87.44%)	1622 (12.53%)	4 (0.03%)	12944
Case 5	16607 (91.65%)	1509 (8.33%)	4 (0.02%)	18120
Case 6	10238 (76.16%)	3188 (23.71%)	17 (0.13%)	13443
Case 7	8676 (84.48%)	1587 (15.45%)	7 (0.07%)	10270
Case 8	11898 (76.08%)	3714 (23.75%)	27 (0.17%)	16622
Case 9	16433 (87.55%)	2312 (12.31%)	25 (0.13%)	18770
Case 10	7937 (68.83%)	3568 (12.32%)	26 (0.23%)	11531
Case 11	3004 (68.49%)	1372 (31.28%)	10 (0.23%)	4386
Case 12	7475 (68.30%)	3462 (31.63%)	8 (0.07%)	10945

Legend: Even among patients with gangliocytic paraganglioma, the distribution of the three characteristic tumor cells varied from case to case. The counts and percentages of tumor cells in 50 random high-power fields from histopathological images in each case are summarized.

**Figure 3:**
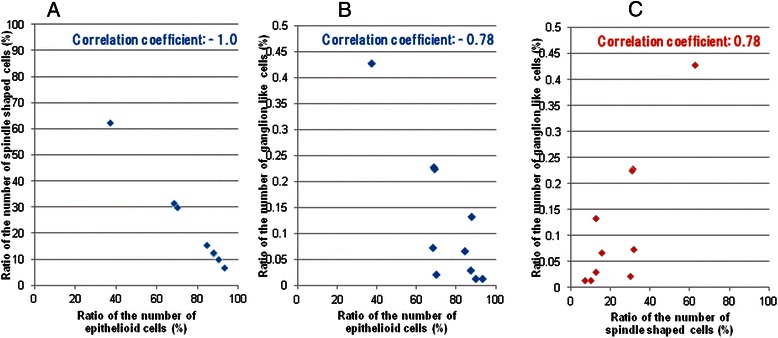
Scatter plots of the ratios of the number of tumor components (epithelioid, spindle-shaped, and ganglion-like cells) in 12 patients with duodenal gangliocytic paraganglioma. Legend: **(A)** A significant negative correlation was found between the percentage of epithelioid and spindle-shaped cells in the 12 patients with gangliocytic paraganglioma. The correlation coefficient was −1.0 (Pearson’s product-moment correlation coefficient; *P* < 0.001). **(B)** A significant negative correlation was found between the percentages of epithelioid to ganglion-like cells in the 12 patients with gangliocytic paraganglioma. The correlation coefficient was −0.78 (Pearson’s product-moment correlation coefficient; *P* < 0.001). **(C)** A significant positive correlation was found between the percentages of spindle-shaped to ganglion-like cells in the 12 patients with gangliocytic paraganglioma. The correlation coefficient was 0.78 (Pearson’s product-moment correlation coefficient; *P* < 0.001).

Additionally, a significant negative correlation was observed between the percentage of epithelioid and spindle-shaped or ganglion-like cells in 12 patients (Figure 3). Conversely, a significant positive correlation was observed between the percentage of spindle-shaped and ganglion-like cells in 12 patients (Figure 3).

### Immunohistochemical examination of gangliocytic paraganglioma

**Figure 4:**
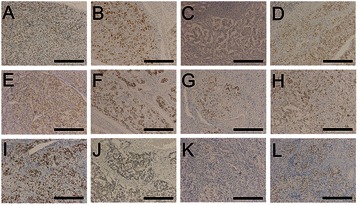
Immunohistochemical reactivity for progesterone receptor in the collected 12 cases of duodenal gangliocytic paraganglioma. Legend: Photomicrographs showing the results of immunohistochemical staining for the progesterone receptor (Panel **A** to **L** represents Case 1 to 12 of gangliocytic paraganglioma). In 11 of the 12 cases examined, epithelioid cells showed positive reactivity for the progesterone receptor (Panel **A** to **L**; immunohistochemistry; magnification: × 200, for each, scale bar represents 300 μm).

**Figure 5:**
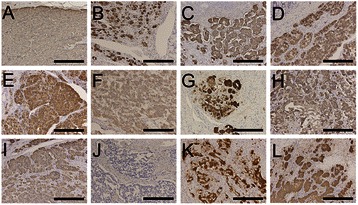
Immunohistochemical reactivity for pancreatic polypeptide in the collected 12 cases of duodenal gangliocytic paraganglioma. Legend: Photomicrographs showing the results of immunohistochemical staining of pancreatic polypeptide (Panel **A** to **L** represents Case 1 to 12 of gangliocytic paraganglioma). In 11 of the 12 cases examined, epithelioid cells showed positive reactivity for pancreatic polypeptide (Panel **A** to **L**; immunohistochemistry; magnification: × 200, for each, scale bar represents 300 μm).

In epithelioid cells, CD56 and synaptophysin showed the highest positive rates (12/12, 100% for both), followed by chromogranin A, pancreatic polypeptide (PP), progesterone receptor, somatostatin (11/12, 91.7% each), and pan-cytokeratins (4/12, 33.3%). In spindle-shaped cells, S-100 protein showed the highest positive rates (12/12, 100%), followed by Bcl-2 (8/12, 66.7%) and CD56 (5/12, 41.7%). In ganglion-like cells, CD56 and synaptophysin showed the highest positive rates (12/12, 100% for both), followed by PP, somatostatin, S-100 protein (11/12, 91.7% each), and chromogranin A (9/12, 75.0%). Additionally, the Ki-67 labeling index of epithelioid, spindle-shaped, and ganglion-like cells ranged from 0.09 to 0.80%, 0.08 to 0.81%, and 0.00%, respectively (Figure 4, Figure 5, Table 3, and Table 4).

**Table 3:** **Immunohistochemical findings of gangliocytic paraganglioma**

	Epithelioid cells	Spindle-shaped cells	Ganglion-like cells
bcl-2	3/12 (25.0%)	8/12 (66.7%)	4/12 (33.3%)
CD56	12/12 (100%)	5/12 (41. 7%)	12/12 (100%)
Chromogranin A	11/12 (91.7%)	0/0 (0%)	9/12 (75.0%)
Estrogen receptor	3/12 (25.0%)	0/0 (0%)	0/0 (0%)
Pancreatic polypeptide	11/12 (91.7%)	0/0 (0%)	11/12 (91.7%)
Pan-cytokeratins	4/12 (33.3%)	0/0 (0%)	0/0 (0%)
Progesterone receptor	11/12 (91.7%)	0/0 (0%)	0/0 (0%)
p53	0/0 (0%)	0/0 (0%)	0/0 (0%)
Somatostatin	11/12 (91.7%)	0/0 (0%)	11/12 (91.7%)
Synaptophysin	12/12 (100%)	3/12 (25.0%)	12/12 (100%)
S-100	1/12 (8.3%)	12/12 (100%)	11/12 (91.7%)

Legend: Results of immunohistochemical examination of the three characteristic tumor components of the collected 12 cases of gangliocytic paraganglioma are summarized.

**Table 4:** **Mitotic activity and Ki-67 immunoreactivity of gangliocytic paraganglioma**

	Epithelioid cells		Spindle-shaped cells		Ganglion-like cells	
cases	Mitotic activity	Ki-67 immunoreactivity	Mitotic activity	Ki-67 immunoreactivity	Mitotic activity	Ki-67 immunoreactivity
1	0 / 1289	7 / 1289 (0.54%)	0 / 1041	3 / 1041 (0.29%)	0 / 4	0 / 4 (0.00%)
2	0 / 1097	5 / 1097 (0.46%)	0 / 1189	4 / 1189 (0.34%)	0 / 4	0 / 4 (0.00%)
3	0 / 1403	6 / 1403 (0.43%)	0 / 1349	2 / 1349 (0.15%)	0 / 103	0 / 103 (0.00%)
4	0 / 1213	5 / 1213 (0.41%)	0 / 1001	6 / 1001 (0.60%)	0/ 7	0/ 7 (0.00%)
5	0 / 1043	4 / 1043 (0.38%)	0 / 1076	3 / 1076 (0.28%)	0 / 4	0/ 4 (0.00%)
6	0 / 1128	9 / 1128 (0.80) %	0 / 1007	2 / 1007 (0.20%)	0 / 31	0 / 31 (0.00%)
7	0 / 1343	4 / 1343 (0.30%)	0 / 1192	3 / 1192 (0.25%)	0/ 38	0/ 38 (0.00%)
8	0 / 1021	3 / 1021 (0.29%)	0 / 1165	3 / 1165 (0.26%)	0 / 71	0 / 71 (0.00%)
9	0 / 1321	3 / 1321 (0.23%)	0 / 1063	2 / 1063 (0.28%)	0 / 48	0 / 48 (0.00%)
10	0 / 1081	2 / 1081 (0.19%)	0 / 1288	1 / 1288 (0.08%)	0 / 46	0 / 46 (0.00%)
11	0 / 1013	1 / 1013 (0.10%)	0 / 1362	11 / 1362 (0.81%)	0 / 12	0 / 12 (0.00%)
12	0 / 1111	1 / 1111 (0.09%)	0 / 1111	1 / 1141 (0.09%)	0/ 14	0/ 14 (0.00%)

Legend: In the present study, epithelioid, spindle-shaped, and ganglion-like cells showed no mitotic activity. In addition, the Ki-67 labeling index of them ranged from 0.09 to 0.80%, 0.08 to 0.81%, and 0.00%, respectively.

### Comparison of gangliocytic paraganglioma with gastrointestinal neuroendocrine tumor grade 1

As previously mentioned, we identified six patients with duodenal NET G1 who were treated at our institute. Among these six patients with duodenal NET G1, patient ages ranged from 61 to 86 years (mean ± SD: 71.2 ± 10.6 years) at diagnosis. Four patients were men and two were women. Two patients underwent an endoscopic procedure and the remainder underwent open surgical resection. The follow-up period ranged from 11 to 87 months and neither recurrence nor deaths in patients with NET G1 were found. Histopathologically, the maximum tumor diameter ranged from 4 to 19 mm (mean ± SD: 6.3 ± 10.2 mm). Four patients had NET G1 localized within the submucosal layer, while two patients had NET G1 invading the muscularis propria. No patients with lymph node metastases were found (Table 5). In immunohistochemistry, CD56, synaptophysin, and chromogranin A showed the highest positive rates (6/6, 100% each), followed by somatostatin (5/6, 83.3%) and pan-cytokeratins (4/6, 66.7%). Duodenal NET G1 tumor cells showed completely negative reactivity for hormone (estrogen and progesterone) receptors and PP. Statistically, GP and NET G1 showed significantly different positive reactivity for progesterone receptor and PP (chi-square test: *P* < 0.05, for both). In the present study, sensitivity and specificity of the progesterone receptor and PP for the histopathological diagnosis of GP were 91.7 and 100%, respectively (Tables 6 and 7).

**Table 5:** **Clinicopathological findings of the collected duodenal neuroendocrine tumor G1**

Cases	Age (years)	Sex	Operation	size (mm)	Depth	Lymph node metastasis	Outcome
1	61	Male	PD	6	sm	Negative	NED 87
2	86	Female	PD	14	sm	Negative	NED 82
3	65	Male	Endoscopic resection	14	mp	Negative	NED 11
4	73	Male	PD	4	sm	Negative	NED 66
5	81	Male	Endoscopic resection	4	sm	Negative	NED 39
6	61	Female	PD	19	mp	Negative	NED 38

*PD*: pancreatoduodenectomy, size: maximum diameter of the tumor, depth: depth of the tumor, sm: submucosal layer, muscularis propria, *NED*: no evidence of disease.

Legend: In this study, a total of six patients with duodenal neuroendocrine tumor (NET) G1 were collected and examined. Clinicopathological findings of these six patients with duodenal NET G1 are summarized.

**Table 6:** **Immunohistochemical findings of duodenal neuroendocrine tumor G1**

	Immunoreactivity in the neuroendocrine tumor G1
bcl-2	0 / 6 (0%)
CD56	6 / 6 (100%)
Chromogranin A	6 / 6 (100%)
Estrogen receptor	0 / 6 (0%)
Pancreatic polypeptide	0 / 6 (0%)
Pan-cytokeratins	4 / 6 (66.7%)
Progesterone receptor	0 / 6 (0%)
p53	0 / 6 (0%)
Somatostatin	5 / 6 (83.3%)
Synaptophysin	6 / 6 (100%)
S-100	0 / 6 (0%)

Legend: Results of immunohistochemical examination of six cases of duodenal neuroendocrine tumor G1 are summarized.

**Table 7:** **Immunohistochemical findings of hormone receptors and pancreatic polypeptide**

	Estrogen receptor	Progesterone receptor	Pancreatic polypeptide
Immunoreactivity in the epithelioid cells of GP	3 / 12 (25.0%)	11 / 12 (91.7%)	11 / 12 (91.7%)
Immunoreactivity in the NET G1	0 / 6 (0%)	0 / 6 (0%)	0 / 6 (0%)
P value (Chi-Square test)	P = 0.180	P < 0.001	P < 0.001

*GP*: Gangliocytic paraganglioma, *NET*: neuroendocrine tumor.

Legend: Duodenal neuroendocrine tumor (NET) G1 except for gangliocytic paraganglioma (GP) showed completely negative reactivity for hormone (estrogen and progesterone) receptors and pancreatic polypeptide. Statistically, GP showed significantly higher positive reactivity for progesterone receptor and pancreatic polypeptide (Chi-square test: *P* < 0.05 for each).

## Discussion

GP is an extremely rare NET that commonly arises in the second part of the duodenum [12,13] and this tumor has often been misdiagnosed as NET G1 given its low cell proliferative activity [1,14-17]. In fact, neither mitosis nor prominent Ki-67 immunoreactivity was found in present GP cases and they met the criteria of typical NET G1. However, since most cases with GP pursue benign course rather than cases with NET G1, it is important to clearly differentiate GP from NET G1. The present study confirmed the presence of a wide spectrum of histopathological findings in the three characteristic tumor components of GP, which is consistent with a previous report [18]. By comparing the immunohistochemical features of GP and NET G1, our study provides information that can be used to differentiate GP from NET G1 with ease. Namely, it was found that epithelioid cells, the major GP component, showed significantly higher positive reactivity for the progesterone receptor and PP (11/12, 91.7% for both) than duodenal NET G1 (0/6, 0%, for both). These findings suggest that confirming reactivity to the progesterone receptor and PP can assist in differentiating GP from NET G1. In particular, we wish to emphasize the importance of confirming PP expression in GP epithelioid cells, because investigators have previously reported a patient with GP showing elevated serum PP [19]. This fact indicates that confirmation of serum PP levels might be a useful marker for monitoring recurrence or metastasis after surgical procedures. Furthermore, mitotic activity and Ki-67 immunoreactivity are prognostic indicators for neuroendocrine tumors [1,4]. However, regardless of whether lymph nodes metastases were present, neither mitotic activity nor prominent Ki-67 immunoreactivity was found in GP cases. Moreover, it has been reported that no mitotic activity was found and Ki-67 labeling index was extremely low both in primary and metastatic foci in a patient who died of GP [8]. These finding suggests that typical prognostic indicators in neuroendocrine tumors may have limited value to evaluate the malignant potential of GP.

The clinicopathological distinction between GP and pancreatic NET is also important. It has been largely accepted that most tumor cells of PP secreting tumors (so called PPoma) show immunoreactivity for PP [20]. However, this tumor commonly arises from the tail of the pancreas [20], whereas the vast majority of GPs arise from the duodenum and only two cases of pancreatic GP have been reported [21,22]. These facts suggest the importance of detailed imaging examinations to differentiate GP from PP secreting tumor. Conversely, previous investigators reported that approximately one-third of pancreatic NETs exhibit PP immunoreactivity [23,24]. This indicates that confirmation of PP immunoreactivity may have some value for differentiating GP from pancreatic NET, except for PP secreting tumor.

Further discussion is warranted regarding the morphometric analysis of GP. To investigate the correlation between the three characteristic components (epithelioid, spindle-shaped, and ganglion-like cells), Pearson’s product-moment correlation coefficients were calculated. If Pearson’s product-moment correlation coefficients had been calculated using tumor cell counts per unit area, the presence of stromal cells could have affected the results. Therefore, Pearson’s product-moment correlation coefficients were calculated in relation to the prevalence of the percentages of these characteristic cell types, rather than the tumor cell counts per unit area. Results showed a negative correlation between the percentage of epithelioid and spindle-shaped or ganglion-like cells. Conversely, a positive correlation was found between the percentage of spindle-shaped and ganglion-like cells. Taken together, these findings suggest that epithelioid GP cells have a different origin from spindle-shaped and ganglion-like cells, as previous investigators reported [10]. Finally, the incidence of GP is worth consideration. GP is regarded as an extremely rare NET; however, the results of this study showed that four (40.0%) of 10 patients with duodenal NET G1 actually had GP. This suggests that GP accounts for a substantial, constant percentage of duodenal NET.

## Conclusions

Standard clinical management of GP has not been established. However, the difference in prognosis between GP and NET emphasizes the importance of differentiating between them. In this study, we showed that immunoreactivity to the progesterone receptor and PP can assist in differentiating GP from NET G1 and we believe that this insight contributes to improving the clinical management of GP.

## Abbreviations

GP: Gangliocytic paraganglioma
NET: Neuroendocrine tumor
PP: Pancreatic polypeptide
WHO: World Health Organization
HPF: High-power field
SD: Standard deviation
